# Recent advances in understanding hematopoiesis in Fanconi Anemia

**DOI:** 10.12688/f1000research.13213.1

**Published:** 2018-01-24

**Authors:** Grover Bagby

**Affiliations:** 1 Departments of Medicine and Molecular and Medical Genetics, Division of Hematology and Medical Oncology, Knight Cancer Institute, Oregon Health & Science University, Portland, Oregon, USA

**Keywords:** Fanconi anemia, hematopoiesis, stem cells, DNA repair, oxidative stress, mitophagy, autophagy, virophagy, inflammation, interferon, tumor necrosis factor, TGF, leukemogenesis, bone marrow failure, aplastic anemia, gene therapy, bone marrow transplantation

## Abstract

Fanconi anemia is an inherited disease characterized by genomic instability, hypersensitivity to DNA cross-linking agents, bone marrow failure, short stature, skeletal abnormalities, and a high relative risk of myeloid leukemia and epithelial malignancies. The 21 Fanconi anemia genes encode proteins involved in multiple nuclear biochemical pathways that effect DNA interstrand crosslink repair. In the past, bone marrow failure was attributed solely to the failure of stem cells to repair DNA. Recently, non-canonical functions of many of the Fanconi anemia proteins have been described, including modulating responses to oxidative stress, viral infection, and inflammation as well as facilitating mitophagic responses and enhancing signals that promote stem cell function and survival. Some of these functions take place in non-nuclear sites and do not depend on the DNA damage response functions of the proteins. Dysfunctions of the canonical and non-canonical pathways that drive stem cell exhaustion and neoplastic clonal selection are reviewed, and the potential therapeutic importance of fully investigating the scope and interdependences of the canonical and non-canonical pathways is emphasized.

## Introduction

Fanconi anemia (FA) is a rare inherited syndrome characterized by progressive bone marrow failure (BMF) and a high relative risk of hematopoietic and epithelial malignancies in children who sometimes have characteristic developmental abnormalities, including short stature and radial ray defects. Since the first clinical report of this disease 90 years ago ^1^, important clinical advances have included the development of the gold-standard diagnostic test (chromosomal breakage responses when lymphocytes or fibroblasts are exposed to low doses of either mitomycin C or diepoxybutane) ^2– 4^ and major improvements in the application of stem cell transplantation to cure the hematopoietic defect in children with FA ^5– 13^. Not all patients have a suitable donor, and successful transplants are not infrequently associated with the subsequent development of solid tumors and other unique ‘late effects’ ^14– 17^, so less toxic forms of therapy are clearly desirable. Their development does, however, require a more complete understanding of molecular pathogenesis than any of us has today. The first hurdle cleared on this point of pathogenesis was the identification of the commonly mutated genes. Since the cloning of the first gene ( *FANCC*) in 1992 ^18, 19^, 20 other genes have been identified ( Table 1) and others may soon be confirmed ^20^. The field of DNA damage repair has advanced substantially as a result of this work, and novel factors that contribute to BMF in FA have emerged. Although this is a rare disorder, it is increasingly serving as a pathophysiological paradigm for more common acquired hematopoietic disorders in patients without FA ^21– 24^.

**Table 1.** Fanconi anemia genes.

Gene	Synonyms	Chromosome	References
*FANCA*		16q24.3	29
*FANCC*		9q22.32	18, 19
*FANCG*	XRCC9	9p13.3	30
*FANCE*		6p21.31	31
*FANCF*		11p14.3	32
*FANCL*		2p16.1	33
*FANCB*	FAAP95	Xp22.2	25
*FANCM*		14q21.1	27, 34
*FANCI*		15q26.1	35, 36
*FANCD2*		3p25.3	37
*FANCD1*	BRCA2	13q13.1	38
*FANCJ*	BRIP1	17q23.2	39, 40
*FANCN*	PALB2	16p12.2	41
*FANCO*	RAD51C	17q22	42
*FANCP*	SLX4	16p13.3	43, 44
*FANCQ*	ERCC4 and XPF	16p13.12	45
*FANCR*	RAD51	15q15.1	46
*FANCS*	BRCA1	17q21.31	47, 48
*FANCT*	UBE2T	1q31.3	49
*FANCU*	XRCC2	7q36.1	50, 51
*FANCV*	REV7 and MAD2L2	1p36.22	52

## Genetics

FA is an autosomal recessive disorder (except those rare cases associated with mutations of *FANCB* located on chromosome X ^25^ and autosomal dominant mutations of FANCR ^26^). For a DNA sequence to be considered an FA gene, an inactivating mutation must be associated with classic chromosomal breakage in response to *ex vivo* challenge with cross-linking agents (mitomycin C or diepoxybutane) in at least one patient and complementation with the wild-type version of the mutant alleles must correct the aberrant chromosomal breakage response. Most patients will have signs of BMF and a high likelihood of developing epithelial malignancies early in life, but few patients (for example, those with *FANCM* mutations) develop malignancies without first showing clinical signs of marrow failure ^27^. Whether the hematopoietic stem cells (HSCs) of *FANCM* patients are truly ‘fit’ is still in question because HSCs in the *FANCM*-deficient murine model have not been stringently tested *in vivo* (for example, using competitive repopulation analysis or exposure to inflammatory stressors) ^28^.

## Stabilizing the genome: the canonical functions of Fanconi anemia proteins

Every FA gene encodes a protein that functions unambiguously to facilitate repair of cross-linked DNA. In response to interstrand crosslinks (ICLs), a core complex of eight proteins (FANCA, -B, -C, -E, -F, -G, -L, and -M) is required ^53, 54^ for monoubiquitinylation of two other proteins (FANCD2 and FANCI) ^53, 35, 55– 57^. This post-translational modification is required for the ‘downstream’ activation of other proteins that unhook ICLs (FANCP and FANCQ) and facilitate homologous recombination (FANCD1, -J, -N, -O, -R, -S, and -U) ^58^. Other nuclear functions of FA proteins in mitosis prevent aneuploidy and aberrant centrosome accumulation ^59– 61^. This review focuses on molecular pathogenesis in FA hematopoietic tissues and addresses the question of whether the canonical functions of the FA proteins account for all of the phenotypic features characteristic of this disease.

## Hematopoietic defects and the prospects of gene therapy

Although clinical marrow failure progresses over time in the lives of patients with FA, it is clear that HSCs are defective early in development. That functionally corrective somatic mutations in a single human HSC prenatally are capable of completely replacing the hematopoietic tissues in twins by the time of birth ^62^ is conclusive proof that the repopulating capacity and overall fitness of the mutant HSCs are unambiguously reduced during development and can be overtaken by even one HSC in which the mutation has been corrected. This conclusion has been confirmed in human embryonic stem cells ^63^ and *Fancc* ^–/–^ and *Fancd2* ^–/–^ mice ^64, 65^. The experiment of nature ^62^ provides clear theoretical evidence that if a single corrected stem cell can so massively outperform all of the mutant ones during development, gene therapy for this disease early in life (or perhaps *in utero* ^66^) can be successful in the future. In fact, many preclinical and a few clinical gene therapy studies have been completed or are ongoing ^67– 72^. Although there have been no clear successes in patients yet ^73^, the goal is within sight and entirely rational.

## Endogenous cross-linking factors

Although hypersensitivity to cross-linking agents remains the *sine qua non* of FA diagnosis, until recently it had not been clear that cross-linking agents, apart from those that might be lurking in the external environment, played a role in the BMF so commonly found in FA. Work by Patel and colleagues has provided some important answers on this point. Having observed that alcohol dehydrogenase-5 (ADH5) deficiency results in synthetic lethality when combined with FA gene suppression in the chicken DT40 cell line ^74^, the group confirmed that loss of either ADH5 ^75^ or aldehyde dehydrogenase-2 (ALDH2) ^76^—enzymes that catabolically inactivate formaldehyde and acetaldehyde, respectively—markedly increases the intensity and onset of severe BMF in *Fancd2*-deficient mice. Given the universal intolerance of FA cells to cross-linking agents (the basis of the gold-standard diagnostic test), it is not surprising that reducing the catalysis of endogenous cross-linking agents would make the disease worse, but what is clear from these studies is that endogenous aldehydes represent major threats to FA-HSCs. Based upon these findings, it was soon discovered that Japanese FA patients with the dominant negative *ALDH2* allele (rs671) have accelerated BMF ^77^. Some investigators have proposed that steady-state endogenous aldehydes themselves account for marrow failure through DNA damage-induced cell death ^78^. This might be true but more experiments need to be done on this score because aldehydes are capable of cross-linking many more types of molecules than just DNA ^79^ and some cross-linked non-DNA targets stimulate two other inter-related processes known to be involved in FA pathogenesis, namely inflammation and oxidative stress ^80– 82^. Autophagosome formation can also be directly inhibited by singlet oxygen-mediated cross-linking of p62, a scaffold protein normally involved in proper autophagosome formation and function ^83^. Moreover, inflammatory processes can suppress the expression of ALDHs in other non-hematopoietic tissues as well as macrophages ^84– 87^. Therefore, in seeking pharmacological remedies for BMF in FA, one needs to consider not only agents that activate or otherwise enhance the function of ALDHs ^88^ but also the basic mechanisms leading to their suppression in the face of inflammation and other stressful cues. It should also be considered that normal FA proteins might enhance the activity of ALDH isoforms directly or indirectly.

## Non-canonical biochemical functions contribute to molecular pathogenesis

The coordinated interactions of FA proteins play an essential role in responding to environmental toxins, and the loss of function of any one of them destabilizes genomes. Because these proteins are so unambiguously essential in nuclear processes that protect and repair the genome, there has been a widespread tendency to simply attribute HSC dysfunction to accrued DNA damage and cell death resulting from the lost canonical functions of the proteins in DNA repair. However, recently, studies have shown that at least some of the FA proteins participate in control of signaling pathways that stress HSCs and the loss of those functions can play a role in disease progression.

Two things have become clear: (1) while all FA proteins play a role in stabilizing the genome, many of them have additional functions ^89– 100^, some of which are independent of other FA proteins or effected outside the nuclear envelope ^90– 93^ and some are functionally independent of the DNA damage response ^102– 104^, and (2) important endogenous factors that suppress FA HSC function are emerging and include proteins normally involved in the inflammatory response ^91, 93, 94, 105– 108^, pathways functionally linked to or activated by inflammation, including oxidative stress ^109– 117^, mitophagy ^102^, and the production of endogenous aldehydes ^74– 77, 118^. Some of the non-canonical functions of FA proteins are listed in Table 2. In light of these disparate functions, it is no surprise that the cellular consequences of FA mutations in HSCs would necessarily include loss of quiescence ^108, 119, 120^, excessive apoptosis when challenged with inflammatory cytokines or redox stress ^110, 121, 122^, and ultimately progressive stem cell attrition in the face of inflammatory stress ^108, 123^.

**Table 2.** Non-canonical functions of Fanconi anemia proteins.

Fanconi anemia proteins	Biochemical function	Expected effects
FANCD2 and FANCA	In response to oxidative stress, FANCD2 and FANCA form a complex with BRG1 within promoters of antioxidant genes ^128^.	Enhance antioxidant defenses
FANCD2	Binds to FOXO3A and reduces reactive oxygen species (ROS) accumulation and enhances antioxidant gene expression ^129^.	Reduces accumulation of ROS and enhances cellular resistance to oxidative stress
FANCG	Binds to and stabilizes mitochondrial PRDX3. Loss of FANCA or FANCC also destabilizes PRDX3 ^98^.	Enhances resistance to resistance to H _2_O _2_ and mitomycin C
FANCC	Binds GSTP1 and activates its activity in response to apoptotic stimuli ^110^.	Prevents apoptosis in growth factor- deprived hematopoietic cells
FANCN	Binds to KEAP1 ^99^	Enhances the oxidative stress response
FANCC, -A, -F, -L, -D1, -D2, and -S	Clear damaged mitochondria (mitophagy) ^102^. FANCA and FANCC interact with Parkin and translocate to damaged mitochondria. Knockdown of FANCC, -F, or -L leads to defective selective autophagy ^101^.	Decrease mitochondrial ROS production ^136^ Reduce activation of inflammasomes ^130^
FANCD2	Localizes to mitochondria and interacts with mitochondrial protein (Atad3) ^100^.	Stabilizes mitochondria, enhances cisplatin resistance, and suppresses apoptosis ^137^
FANCP	Using different domains, FANCP interacts with XPF and ERCC1 to repair interstrand crosslinks and interacts with MUS81 to resist TOP1 inhibitors ^89^.	Mediates resistance to both cross-linking agents and topoisomerase I inhibitors
FANCD2 and FANCA	Suppress transforming growth factor beta signaling in hematopoietic stem and progenitor cells (HSPCs) in the ground state and during inflammatory stress and enhance expression of genes involved in homologous recombination ^94^.	Enhance HSPC survival and function in the face of inflammation and cross-linking agents
FANCC	Binds hsp70 and suppresses the kinase activity of PKR independently of the core complex ^91, 93^.	Enhances survival of cells exposed to inflammatory cytokines
FANCC	Binds to and facilitates activation of STAT1 in response to hematopoietic growth factors ^90, 92^.	Facilitates hematopoietic growth factor signaling
FANCC and FANCA	Suppress Toll-like receptor (TLR)-induced tumor necrosis factor alpha ( *TNFα*) and interleukin 1 beta ( *IL-1β*) expression in macrophages ^130, 132^.	Prevent overproduction of inflammatory cytokines in macrophages
FANCC	Complexes with CtBP1 and suppresses *DKK1* (a *WNT* suppressor) gene expression ^133^.	Facilitates WNT signaling and hematopoietic stem cell self-renewal
FANCL	Ubiquitinates beta-catenin, enhancing its nuclear function ^134^.	Enhances pluripotency of HSPCs
FANCP	Suppresses accumulation of cytoplasmic DNA and the consequent activation of the interferon pathway ^135^.	Suppresses cGAS-STING and reduces pro-inflammatory cytokine production induced by replication stress
FANCD2 and FANCI	Associate with the spliceosomal protein SF3B1 independently of the core complex and influence SF3B1 trafficking ^97^.	Enhance stem cell function ^138^, coordinate DNA replication and co-transcriptional processes, and likely enhance erythropoiesis

## Inflammation: a damaging stressor in Fanconi anemia hematopoietic cells

Because of the known myelosuppressive role of interferon gamma (IFNγ) in the pathogenesis of acquired aplastic anemia ^124– 126^, early *in vitro* studies on FA cells were conducted to test the idea that FA cells might somehow be hypersensitive to this and other known inhibitory cytokines (for example, tumor necrosis factor [TNF]). These studies were positive ^105, 106, 127^ and led to the identification of some non-canonical signaling pathways with which FA proteins were directly involved ^91, 93^. These early studies were conducted *in vitro*, but more recent work has unambiguously confirmed that murine FA stem cells are uniquely vulnerable to inflammatory stresses of many different kinds ^108, 123^ and that, in one informative model, blocking the function of the inflammatory cytokine interleukin 1 beta (IL-1β) (a suppressor of FA HSC proliferation *in vitro*) ^130^ prevented marrow failure ^123^. Multiple inflammatory cytokines and adhesion molecules are involved in the inflammatory response, including factors that can enhance and others that inhibit the replicative capacity of hematopoietic stem and progenitor cells (HSPCs), so it will be important to sort out whether the ‘replicative stress’ induced in the FA HSPC pool plays a role in damaging the cells or whether the suppressive and pro-apoptotic and differentiation-inducing effects of inflammatory cytokines cause them to perish. But the inflammatory response and its association with oxidative stress (to which FA cells are also hypersensitive ^128, 129, 139– 141^) provide opportunities for pharmacological intervention either in the ground state or during episodes of overt inflammation.

A variety of complex indirect effects likely account for some of the inflammatory vulnerabilities of FA HSCs. For example, wild-type FANCC interacts with hsp70 to suppress activation of eukaryotic translation initiation factor 2-alpha kinase 2 (PKR) in wild-type cells exposed to IFNγ and double-stranded RNA or IFN and TNF. The interaction involves the canonical substrate-binding domain of HSP70, and loss of the FANCC/hsp70 interaction results in hyperactivation of PKR in FA cells ^91, 93, 107^. Interestingly, mutations of FANCA, FANCG, and FANCC enhance the binding interaction of FANCC and PKR and result in PKR hyperactivation as well ^142^. The recently described differential FANCA-binding functions of HSP90 and HSP70 and the functional consequences of such differential binding on the canonical function of various FANCA mutant substrates ^143^ add to the complexity of the future *in vivo* studies now required to confirm that these effects are truly independent of induced DNA damage. Finally, that ALDH and hsp70 are induced by and collaborate to control certain viral infections ^144^ suggests that the relationships between ALDH, heat shock proteins, IFN-dependent pathways, and FA proteins should also be formally examined because they may be perturbed interdependently in FA cells.

### Macrophage dysfunction fans the flames

One of the additional challenges associated with the aberrant FA inflammatory response is that FA mononuclear phagocytes, the progeny of FA HSCs, overproduce such cytokines in response to Toll-like receptor (TLR) activation ^104, 130, 132– 145^. So the pharmacological suppression of induced cytokine production or function may also reduce the stress on the HSPC pool. Some preliminary *in vitro* evidence of this had been reported ^145^, but the scope of non-canonical signaling defects in FA is increasingly broad and involves interdependent pathways that pose a challenge for picking a particular molecular focus. Cross-talking environmental cues that induce inflammation, for example, regularly induce oxidative stress ^146– 149^ and enhance endogenous aldehyde loads ^82, 84, 86^. Any one of these stressors can excessively suppress FA HSPCs and induce hyperactive inflammatory cytokine production in more well-differentiated myeloid cells.

### Mitophagic defects

Some FA proteins now provide novel functional insights on long-recognized ties between inflammation and oxidative stress. Many of the non-canonical functions outlined in Table 2 have to do with controlling oxidative stress. For example, FANCC and FANCA normally participate in mitophagic responses by binding to Parkin (which itself is known to play a role in removing damaged mitochondria ^150^), thereby clearing damaged mitochondria and reducing reactive oxygen species. Knockdown of *FANCC*, *-F*, or - *L* also leads to defective mitophagy ^101^. Importantly, the function of FANCC in Parkin-mediated mitophagy is independent of its role in genomic DNA damage repair ^102^. This point was recently demonstrated by using a *FANCC* mutant which does not rectify the dysfunction induced by FA mutations in the canonical pathway but does correct the Parkin-mediated mitophagic function of the protein ^102^. This same *FANCC* mutant (c.67delG, also known as 322delG) also rescues defective STAT1 activation in *FANCC*-deficient cells without correcting the canonical pathway ^90, 92^ and is reportedly associated with a milder phenotype ^151^, suggesting that parallel non-canonical pathways contribute to progression of BMF in FA.

## Experimental therapeutics

In small-molecule therapy of FA, target candidates could include hyperactive signaling pathways in mutant cells that include p53 and p21 ^152^, PKR ^91^, p38 MAPK ^130, 141^, the TLR pathway (p38 MAPK and MK2) ^130, 132^, and, most recently, hyperactive transforming growth factor beta (TGFβ) signaling ^94^. The ideal therapeutic agent would be one that *in vivo*: (1) reduces chromosomal breakage of all FA somatic cells exposed to endogenous and exogenous cross-linking agents, (2) enhances survival of FA cells exposed to genotoxic agents, (3) relieves the defects in FA HSCs in the ground state and in the face of inflammatory challenges, (4) reduces overproduction of or aberrant responses to reactive oxygen species, (5) enhances the metabolic inactivation of endogenous aldehydes, and (6) relieves the overproduction of inflammatory cytokines in more well-differentiated cells (hematopoietic and otherwise). In fact, the TGFβ ‘pathway’ results in many of these favorable responses in FA cells ^94^, so it is currently an appealing target.

### Transforming growth factor beta signaling

Antibody-mediated inhibition of the myelosuppressive protein TGFβ enhanced survival and performance of murine *Fancd2* ^–/–^ HSCs, increased the percentage of quiescent HSPCs in *Fancd2*-deficient mice after exposure to polyinosinic:polycytidylic acid (pI:pC), and prevented pI:pC-induced marrow failure. Interestingly, the titer of DNA damage in mutant HSPCs was also reduced by the treatment with the antibody, a finding that led to the discovery that TGFβ inhibition enhanced homologous recombination repair of double-strand breaks (more likely to be error-free ^153^) and decreased non-homologous end joining (error prone ^153^) in FA cells and the confirmation that the same effect could be achieved by using a small-molecule inhibitor (SD208) ^94^. Why the TGFβ pathway is hyperactivated in FA cells is unclear, so a number of important questions remain to be addressed, including the potential impact on induced cytokine production in FA auxiliary cells (for example, macrophages) and on expression of various ALDHs in both auxiliary cells and HSCs. The role of non-SMAD signaling pathways ^154, 155^ ought to be tested, particularly since some of them are known to be involved in TLR hyper-responsiveness in FA macrophages ^130, 131^ and in suppression of FA erythropoiesis *in vitro* ^145^. Paracrine and autocrine sources of such factors must be identified and HSPC subtypes need to be carefully investigated because responses to TGFβ pathway disruption of various subsets can differ substantially ^156^. Ruling out functional interference with other molecules of the 33-member TGFβ superfamily ^157^ (for example, BMP-6) ^158, 159^ would help address safety concerns for any candidate molecule as would consideration of the anti-oncogenic function of TGFβ1 in modulating the transforming activity of HOXA9 ^160^. This latter point is essential given the known high risk in FA patients of clonal evolution to myelodysplastic syndrome or acute myeloid leukemia. Nonetheless, although more work is required, the identification of the TGFβ pathway as a potential therapeutic target is buttressed by having met more standards for pharmacological candidacy than any other small-molecule approach to date.

## Evolution and clonal selection

Loss of the non-canonical functions of the FA proteins likely serves to exacerbate or activate processes that result in HSC dysfunction. Involvement of these proteins in protection from oxidative stress, inflammatory cues, and endogenous aldehydes and in facilitating selective autophagic responses occurs, at least in part, by way of *parallel* pathways that have evolved to protect the genome and protect germ cells ^106, 161, 162^ and HSCs. This makes evolutionary sense. In multicellular organisms, this would simply ensure that loss of FA protein function would be attended by loss of fitness in the stem cell pool, limiting the rapid emergence of mutants. Unfortunately, in patients with FA, loss of HSC fitness leads most frequently to life-threatening BMF.

In nature, three things can happen to FA HSCs and two of them are undesirable. HSCs take the one good path least commonly. Here, a single FA HSC can mutate in a way that causes reversion of the mutation on one of the FA alleles (known as mosaicism ^163– 165^). In some cases in which mosaicism occurred *in utero*, the progeny of one mutant stem cell replaced the entire hematopoietic systems of twins, neither of whom ever developed BMF ^62^. The second pathway, stem cell death and stem cell exhaustion, leads to the first of two undesirable outcomes, BMF. This is the most common early life-threatening complication in patients with FA. The third pathway is the selection of neoplastic stem cell clones. In this process, the loss of HSC fitness in the face of such an unstable genome creates an opportunity for selection of stem cell clones that have gained, through *somatic* mutations, a new capacity to either exploit or resist the very factors that had previously controlled their state of unfitness.

For example, FA hematopoietic progenitor cells are characteristically suppressed by TNF and IFNγ *in vitro* during the non-clonal aplastic phase have been seen to proliferate abundantly in response to IFNγ and TNF *in vitro* during the myelodysplastic (clonal) phase ^23^. This process has also been confirmed in FA mice. Specifically, exposure of *Fancc*-deficient HSPCs to both growth factors and the inhibitory factor TNF *in vitro* suppressed their growth compared with the growth of wild-type cells, but over time, in some cases, rapid *in vitro* growth of FA-deficient cells ensued. The rapidly proliferating cells had clonal cytogenetic defects and had acquired resistance to TNF. When transplanted into congenic wild-type mice, they induced fatal acute myelogenous leukemia ^166^. The canonical pathway in these surviving leukemic cells was still fully defective but they had gained a survival advantage by ‘quasi-adaptively’ developing cytokine resistance.

The idea of multifunctionality of some of the FA proteins has met with some resistance in large part because the non-canonical processes affected can be either the cause of DNA damage or a consequence of it. To those who would argue that non-canonical pathways play a minor role or no role at all in reducing fitness of FA HSCs and that all non-canonical functions of FA proteins described to date are simply downstream consequences of DNA damage, I would raise two points. First, the natural selection of ‘adapted’ stem cell clones serves as evidence that non-canonical pathways *are* involved and contribute in a major way to the selection of neoplastic clonal hematopoiesis. Second, it is worth re-emphasizing that the murine models of FA develop truly fulminant BMF only when an inflammatory response is provoked *in vivo* ^108, 123^, so even if the non-canonical functions play a minor role, interdiction of the non-canonical phenomena that provoke stem cell attrition should have therapeutic value. In fact, Eklund’s group has shown that this is the case at least in FA mice ^123^. These observations have created some enthusiasm for attacking the molecular basis of stress intolerances in FA cells. They also create hope that by reducing selective pressures and selective inflammatory sweeps through the FA HSC pool, BMF could be prevented or forestalled and neoplastic clones would not emerge.

In summary, the advances in the FA field are exciting. They have resulted not only from a focus of the research community on the biochemistry of DNA damage repair but from an increasing recognition that the FA proteins are multifunctional and participate directly in biochemical pathways that effect protective responses to endogenous aldehydes, oxidative stress, inflammation, mitophagy, and virophagy. This increasingly complex expansion of the FA field may lead to more holistic clarity on molecular pathogenesis not only of BMF, leukemogenesis and cancer but of skeletal and cutaneous manifestations of this disease. Finally, today we recognize that this disease provides a uniquely informative window through which we can view more clearly the potential role of selective sweeps in the pathogenesis of myeloid neoplasms in the general population ^21, 22, 167^.
